# Miscellany

**Published:** 1908-06

**Authors:** 


### MISCELLANY

The United States Civil Service Commission announces an examination on June 17, 1908, at the usual places, to secure eligibles from which to make certification to fill a vacancy in the position of dental interne (male), at $600.00 per annum, with maintenance, in the Government Hospital for the Insane, Washington, D. C., and vacancies requiring similar qualifications as they may occur. The Department states that it reserves the right to terminate the appointment at the expiration of one year of service if it is deemed advisable to do so.

Applications will not be accepted from persons who fail to indicate, in answer to question 17 of the application form, that they are graduates of not more than 18 months' standing of regularly incorporated dental colleges. Applicants must be unmarried. Age limit, 20 years or over on the date of examination. This examination is open to all citizens of the United States who comply with the requirements.

---

The United States Civil Service Commission announces an examination on July 1, 1908, to secure eligibles from which to make certification to fill a vacancy in the position of technical assistant, Division of Pharmacology, Hygienic Laboratory, Public Health and Marine-Hospital Service, at $150 per month, and vacancies requiring similar qualification as they may occur in any branch of the service.

Applicants should have received either the degree of M.D. or Ph.D. from institutions of high standing. If the former, they should also have had hospital experience or exceptional opportunities of pursuing graduate studies; if the latter, their studies should have been along the lines indicated above. These facts must be fully stated in the application.

